# 4-Hy­droxy-6-methyl­pyridin-2(1*H*)-one

**DOI:** 10.1107/S1600536813024240

**Published:** 2013-09-12

**Authors:** Héctor Reyes, Gerardo Aguirre, Daniel Chávez

**Affiliations:** a Centro de Graduados e Investigación del Instituto Tecnológico de Tijuana, Apdo. Postal 1166, 22500, Tijuana, B.C., Mexico

## Abstract

In the crystal structure of the title compound, C_6_H_7_NO_2_, N—H⋯O and O—H⋯O hydrogen bonds link the mol­ecules, forming a zigzag array along [001] and a layer structure parallel to the *ab* plane.

## Related literature

For the potential of related compounds in anti-HIV treatment, see: De Clercq (2005 ▶); Dollé *et al.* (1995 ▶); Medina-Franco *et al.* (2007 ▶).
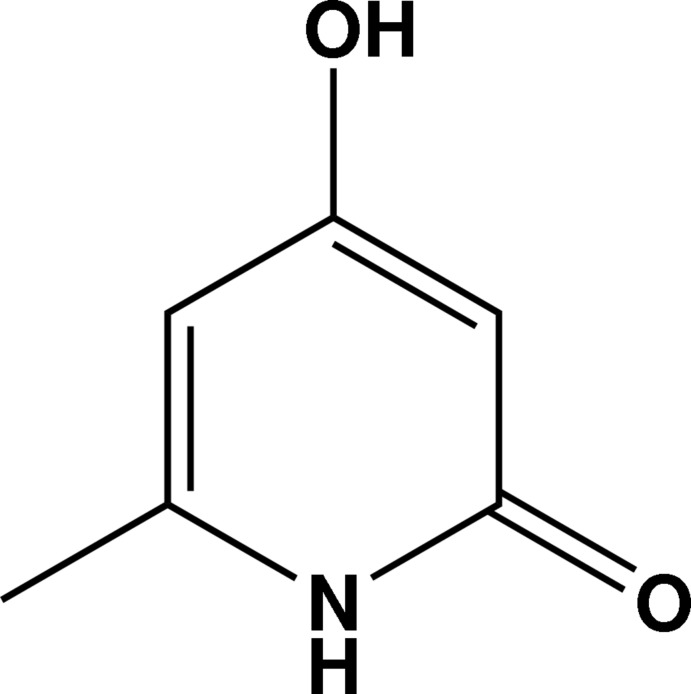


## Experimental

### 

#### Crystal data

C_6_H_7_NO_2_
*M* *_r_* = 125.13
Monoclinic, 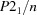

*a* = 4.7082 (5) Å
*b* = 12.2988 (8) Å
*c* = 10.0418 (7) Å
β = 91.303 (7)°
*V* = 581.32 (8) Å^3^
*Z* = 4
Mo *K*α radiation
μ = 0.11 mm^−1^
*T* = 298 K
0.65 × 0.20 × 0.18 mm

#### Data collection

Bruker P4 diffractometer
Absorption correction: ψ scan (*XSCANS*; Siemens, 1996 ▶) *T*_min_ = 0.216, *T*_max_ = 0.259
2445 measured reflections
1701 independent reflections
1269 reflections with *I* > 2σ(*I*)
*R*_int_ = 0.026
3 standard reflections every 97 reflections intensity decay: 9.4%

#### Refinement

*R*[*F* ^2^ > 2σ(*F* ^2^)] = 0.050
*wR*(*F* ^2^) = 0.160
*S* = 1.06
1701 reflections
82 parameters
H-atom parameters constrained
Δρ_max_ = 0.32 e Å^−3^
Δρ_min_ = −0.25 e Å^−3^

### 

Data collection: *XSCANS* (Siemens, 1996 ▶); cell refinement: *XSCANS*; data reduction: *XSCANS*; program(s) used to solve structure: *SHELXS97* (Sheldrick, 2008 ▶); program(s) used to refine structure: *SHELXL97* (Sheldrick, 2008 ▶); molecular graphics: *SHELXTL* (Sheldrick, 2008 ▶); software used to prepare material for publication: *SHELXTL*.

## Supplementary Material

Crystal structure: contains datablock(s) I. DOI: 10.1107/S1600536813024240/im2437sup1.cif

Structure factors: contains datablock(s) I. DOI: 10.1107/S1600536813024240/im2437Isup2.hkl

Supplementary material file. DOI: 10.1107/S1600536813024240/im2437Isup3.cml

Additional supplementary materials:  crystallographic information; 3D view; checkCIF report

## Figures and Tables

**Table 1:** Hydrogen-bond geometry (Å, °)

*D*—H⋯*A*	*D*—H	H⋯*A*	*D*⋯*A*	*D*—H⋯*A*
N1—H1*A*⋯O1^i^	0.86	1.98	2.835 (2)	175
O2—H2*B*⋯O1^ii^	0.82	1.79	2.609 (2)	180

Symmetry codes: (i) 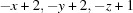; (ii) 
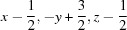
.

**Fig. 1.**
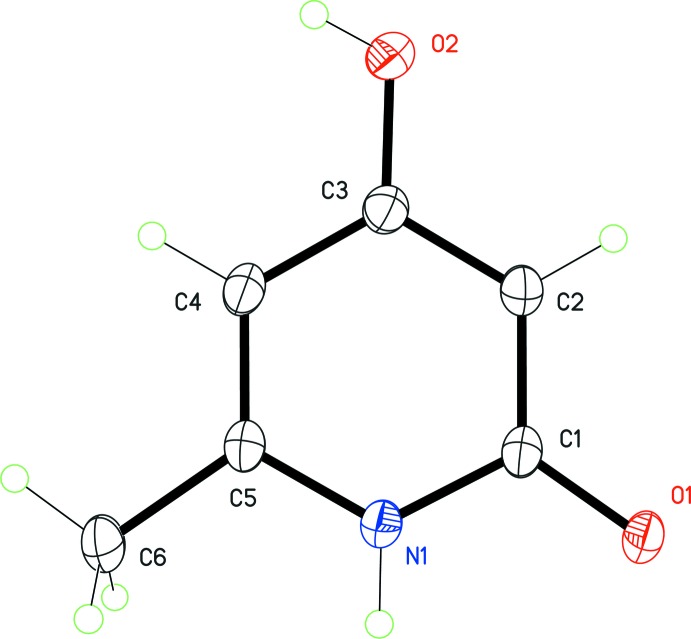
Molecular structure of the title compound with displacement ellipsoids drawn at the 30% probability level.

**Fig. 2.**
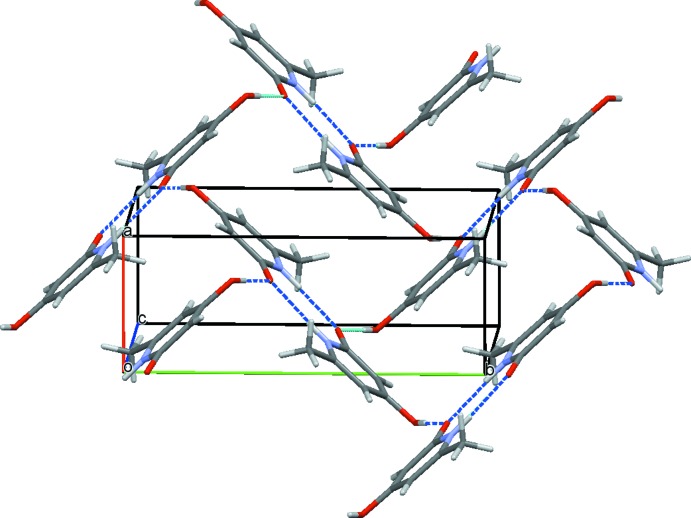
Hydrogen-bond (indicated as dashed lines) network of the title compound leading to a two dimensional network along the ab plane.

## supplementary crystallographic information

### 1. Comment

The acquired immunodeficiency syndrome (AIDS) is a disease of people who are infected with human immunodeficiency virus (HIV). The use of drugs to fight HIV are called antiretroviral drugs and are characterized by inhibiting essential enzymes for virus replication, such as reverse transcriptase (De Clercq, 2005).

Pyridin-2 (1*H*)-one hybrids are a kind of compounds that inhibit the reverse transcription process and have been shown, by molecular modeling, their good performance as non-nucleoside inhibitors of HIV-1 reverse transcriptase. Recent design of the pyridin-2(1*H*)-one hybrids has generated active molecules against wild type and mutant strains of HIV, as in the case of second-generation hybrid pyridinone-UC781 (Medina-Franco *et al.*., 2007). In this work, and as part of our ongoing research, we have synthesized pyridin-2 (1*H*)-one hybrids (Dollé *et al.*, 1995) of second generation with different polar groups at C-3 and also with different olefinic groups at C-4, similar to pyridinone-UC781. The compound 4-hydroxy-6-methylpyridin-2 (1*H*)-one is an intermediate in the synthesis of second-generation hybrids with a polar nitro group at C-3.

We have synthesized the title compound (I) and report its crystal structure here (Fig. 1). In the crystal structure adjacent networks are linked together *via* intermolecular hydrogen bond interactions (table 1) [N1—H1*A*···O1^i^ (2.8349 Å), symmetry codes: (i) –x + 2, –y + 2, - *z* + 1] and [O2—H2*B*···O1^ii^ (2.6086 Å), symmetry codes: (ii) *x* – 1/2, - *y* + 3/2, *z* – 1/2] to form a zigzag array along the [001] direction and molecules are forming a layer structure parallel to the *ab* plane (Fig. 2).

### 2. Experimental

The synthesis of 4-hydroxy-6-methylpyridin-2(1*H*)-one includes reagents and reagent grade solvents, which were used without further purification. In a round bottom flask of 500 ml equipped with a magnetic stirrer was placed 10.0 g of ethyl 4-hydroxy-6-methyl-2-oxo-1,2-dihydropyridine-3-carboxylate (0.05 mol) in 350 ml of hydrochloric acid 1 N. The mixture was stirred at reflux for 72 h. 4-Hydroxy-6-methylpyridin-2(1*H*)-one precipitated as a white solid (6.2 g, 99%, m. p. 273–275 °C, for analytical data, see _exptl_special_details section). Crystals of the title compound suitable for Xray diffraction were obtained by dissolving 100 mg of 4-hydroxy-6-methyl-pyridine-2 (1*H*)-one in 10 ml of methanol-diethylether (1:1, v / v) and placing the solution in a glass vial. The solution was allowed to stand at room temperature for 7 days and the crystals formed were filtered.

### 3. Refinement

All H atoms were positioned geometrically and refined using a riding model, with C—H = 0.93 Å for aryl and 0.96 Å for methyl H atoms. Isotropic thermal parameters were fixed to U_iso_(H) = 1.2 U_eq(_C) for aryl and U_iso_(H) = 1.5 U_eq_(C) for methyl H atoms.

### Crystal data

**Table:**

C_6_H_7_NO_2_	*F*(000) = 264
*M**_r_* = 125.13	*D*_x_ = 1.430 Mg m^−^^3^
Monoclinic, *P*2_1_/*n*	Mo *K*α radiation, λ = 0.71073 Å
Hall symbol: -P 2yn	Cell parameters from 51 reflections
*a* = 4.7082 (5) Å	θ = 6.6–12.3°
*b* = 12.2988 (8) Å	µ = 0.11 mm^−^^1^
*c* = 10.0418 (7) Å	*T* = 298 K
β = 91.303 (7)°	Prismatic, colorless
*V* = 581.32 (8) Å^3^	0.65 × 0.20 × 0.18 mm
*Z* = 4	

### Data collection

**Table:**

Bruker P4 diffractometer	1269 reflections with *I* > 2σ(*I*)
Radiation source: fine-focus sealed tube	*R*_int_ = 0.026
Graphite monochromator	θ_max_ = 30.0°, θ_min_ = 2.6°
2θ/ω scans	*h* = −1→6
Absorption correction: ψ scan (*XSCANS*; Siemens, 1996)	*k* = −1→17
*T*_min_ = 0.216, *T*_max_ = 0.259	*l* = −14→14
2445 measured reflections	3 standard reflections every 97 reflections
1701 independent reflections	intensity decay: 9.4%

### Refinement

**Table:**

Refinement on *F*^2^	Primary atom site location: structure-invariant direct methods
Least-squares matrix: full	Secondary atom site location: difference Fourier map
*R*[*F*^2^ > 2σ(*F*^2^)] = 0.050	Hydrogen site location: inferred from neighbouring sites
*wR*(*F*^2^) = 0.160	H-atom parameters constrained
*S* = 1.06	*w* = 1/[σ^2^(*F*_o_^2^) + (0.1*P*)^2^] where *P* = (*F*_o_^2^ + 2*F*_c_^2^)/3
1701 reflections	(Δ/σ)_max_ < 0.001
82 parameters	Δρ_max_ = 0.32 e Å^−^^3^
0 restraints	Δρ_min_ = −0.25 e Å^−^^3^

### Special details

**Table:**

Experimental. IR: 3296, 3094, 2891, 1640 cm^-1. 1Ĥ NMR (CDCl~3~): δ 10.99 (s, NH-1), 10.40 (s, OH), 5.59 (s, H-3), 5.34 (s, H-5) 2.07 (s, 3H, CH~3~—C-6). ^13Ĉ NMR (CDCl~3~): δ 167.6, 164.8, 145.9, 98.2, 95.7, 18.5. MS *m*/*e* (int. *rel*): [*M*]^+^ 125 (100), 97 (16).
Geometry. All e.s.d.'s (except the e.s.d. in the dihedral angle between two l.s. planes) are estimated using the full covariance matrix. The cell e.s.d.'s are taken into account individually in the estimation of e.s.d.'s in distances, angles and torsion angles; correlations between e.s.d.'s in cell parameters are only used when they are defined by crystal symmetry. An approximate (isotropic) treatment of cell e.s.d.'s is used for estimating e.s.d.'s involving l.s. planes.
Refinement. Refinement of *F*^2^ against ALL reflections. The weighted *R*-factor *wR* and goodness of fit *S* are based on *F*^2^, conventional *R*-factors *R* are based on *F*, with *F* set to zero for negative *F*^2^. The threshold expression of *F*^2^ > σ(*F*^2^) is used only for calculating *R*-factors(gt) *etc*. and is not relevant to the choice of reflections for refinement. *R*-factors based on *F*^2^ are statistically about twice as large as those based on *F*, and *R*- factors based on ALL data will be even larger.

### Fractional atomic coordinates and isotropic or equivalent isotropic displacement parameters (Å^2^)

**Table:**

	*x*	*y*	*z*	*U*_iso_*/*U*_eq_	
O1	0.7844 (3)	0.91089 (9)	0.60053 (9)	0.0364 (3)	
O2	0.2045 (2)	0.68616 (10)	0.32761 (10)	0.0396 (3)	
H2B	0.2292	0.6556	0.2562	0.059*	
N1	0.7786 (3)	0.93124 (10)	0.37596 (11)	0.0301 (3)	
H1A	0.9049	0.9813	0.3858	0.036*	
C1	0.6811 (3)	0.88072 (12)	0.48782 (12)	0.0288 (3)	
C2	0.4786 (3)	0.79830 (13)	0.46911 (13)	0.0326 (3)	
H2A	0.4026	0.7638	0.5426	0.039*	
C3	0.3912 (3)	0.76793 (12)	0.34229 (13)	0.0303 (3)	
C4	0.4958 (3)	0.82420 (12)	0.23072 (14)	0.0321 (3)	
H4A	0.4353	0.8053	0.1450	0.038*	
C5	0.6858 (3)	0.90624 (12)	0.25012 (13)	0.0293 (3)	
C6	0.8049 (4)	0.97389 (15)	0.14088 (15)	0.0403 (4)	
H6D	0.7267	0.9501	0.0567	0.060*	
H6A	1.0078	0.9662	0.1411	0.060*	
H6B	0.7566	1.0488	0.1548	0.060*	

### Atomic displacement parameters (Å^2^)

**Table:**

	*U*^11^	*U*^22^	*U*^33^	*U*^12^	*U*^13^	*U*^23^
O1	0.0491 (7)	0.0370 (6)	0.0227 (5)	−0.0064 (5)	−0.0062 (4)	0.0009 (4)
O2	0.0483 (7)	0.0407 (6)	0.0297 (5)	−0.0133 (5)	0.0024 (5)	−0.0057 (4)
N1	0.0360 (6)	0.0315 (6)	0.0227 (5)	−0.0037 (5)	−0.0032 (5)	0.0022 (4)
C1	0.0352 (7)	0.0297 (7)	0.0214 (6)	0.0034 (6)	−0.0013 (5)	0.0010 (5)
C2	0.0399 (8)	0.0340 (7)	0.0238 (6)	−0.0026 (6)	0.0015 (5)	0.0009 (5)
C3	0.0324 (7)	0.0311 (7)	0.0273 (6)	0.0002 (6)	0.0000 (5)	−0.0014 (5)
C4	0.0380 (8)	0.0357 (8)	0.0224 (6)	0.0007 (6)	−0.0021 (5)	−0.0023 (5)
C5	0.0341 (7)	0.0319 (7)	0.0218 (6)	0.0027 (6)	−0.0004 (5)	0.0016 (5)
C6	0.0534 (10)	0.0420 (9)	0.0255 (6)	−0.0049 (8)	0.0007 (6)	0.0068 (6)

### Geometric parameters (Å, º)

**Table:**

O1—C1	1.2768 (16)	C2—H2A	0.9300
O2—C3	1.3418 (18)	C3—C4	1.415 (2)
O2—H2B	0.8200	C4—C5	1.359 (2)
N1—C5	1.3629 (17)	C4—H4A	0.9300
N1—C1	1.3719 (17)	C5—C6	1.496 (2)
N1—H1A	0.8600	C6—H6D	0.9600
C1—C2	1.401 (2)	C6—H6A	0.9600
C2—C3	1.3809 (19)	C6—H6B	0.9600
			
C3—O2—H2B	109.5	C5—C4—C3	119.30 (13)
C5—N1—C1	123.42 (13)	C5—C4—H4A	120.4
C5—N1—H1A	118.3	C3—C4—H4A	120.4
C1—N1—H1A	118.3	C4—C5—N1	119.76 (13)
O1—C1—N1	117.79 (14)	C4—C5—C6	124.34 (13)
O1—C1—C2	124.99 (13)	N1—C5—C6	115.90 (14)
N1—C1—C2	117.21 (12)	C5—C6—H6D	109.5
C3—C2—C1	120.46 (13)	C5—C6—H6A	109.5
C3—C2—H2A	119.8	H6D—C6—H6A	109.5
C1—C2—H2A	119.8	C5—C6—H6B	109.5
O2—C3—C2	119.01 (13)	H6D—C6—H6B	109.5
O2—C3—C4	121.24 (12)	H6A—C6—H6B	109.5
C2—C3—C4	119.74 (14)		
			
C5—N1—C1—O1	179.84 (14)	O2—C3—C4—C5	−179.71 (13)
C5—N1—C1—C2	1.0 (2)	C2—C3—C4—C5	1.3 (2)
O1—C1—C2—C3	−176.62 (15)	C3—C4—C5—N1	1.8 (2)
N1—C1—C2—C3	2.2 (2)	C3—C4—C5—C6	−178.05 (15)
C1—C2—C3—O2	177.69 (13)	C1—N1—C5—C4	−3.0 (2)
C1—C2—C3—C4	−3.3 (2)	C1—N1—C5—C6	176.88 (14)

### Hydrogen-bond geometry (Å, º)

**Table:**

*D*—H···*A*	*D*—H	H···*A*	*D*···*A*	*D*—H···*A*
N1—H1*A*···O1^i^	0.86	1.98	2.835 (2)	175
O2—H2*B*···O1^ii^	0.82	1.79	2.609 (2)	180

Symmetry codes: (i) −*x*+2, −*y*+2, −*z*+1; (ii) *x*−1/2, −*y*+3/2, *z*−1/2.
